# Enhanced recovery of terminal sequences of viral RNAs via fine-tuning of the high-throughput sequencing library preparation and evidence of a non-templated nucleotide addition at the 3′ end of the minus strand in members of the genus Ilarvirus

**DOI:** 10.1099/jgv.0.002331

**Published:** 2026-09-03

**Authors:** Dennis Knierim, Paolo Margaria

**Affiliations:** 1 Plant Virus Department, Leibniz Institute DSMZ-German Collection of Microorganisms and Cell Cultures GmbH, Inhoffenstrasse 7B, 38124 Braunschweig, Germany

**Keywords:** high-throughput sequencing, *Ilarvirus*, RACE, RNA ends, RNA tailing

## Abstract

High-throughput sequencing (HTS) technologies have transformed life sciences by enabling rapid, large-scale analysis of nucleic acids, providing unprecedented insights into genomics and transcriptomics. Accurate determination of RNA termini remains, however, a major challenge. We have addressed this limitation by fine-tuning a commercial HTS library preparation protocol. The optimized strategy was validated on plant virus isolates representing diverse taxonomic groups and genome architectures and benchmarked against conventional RACE, demonstrating its effectiveness and robustness. Investigations in members of the genus *Ilarvirus* allowed to prove the occurrence of non-templated nucleotide additions at the 3′ end of the minus-strand viral RNAs *in vivo*, expanding previous results obtained *in vitro* in related members in the *Bromoviridae* and supporting that this feature may be common across the family.

**Impact Statement**

We developed HTS-RARE (High-Throughput Sequencing-Rapid Analysis of RNA Ends), an optimized library preparation strategy that enhances sequencing coverage of RNA termini and enables accurate determination of 5′- and 3′ ends directly from HTS data. The procedure provides a rapid and reliable alternative to labour-intensive RACE methods, particularly benefiting the analysis of segmented viruses and complex samples. Validation across diverse plant RNA viruses demonstrated its broad applicability. Application of HTS-RARE to members of the genus *Ilarvirus* (family *Bromoviridae*) revealed an extra, non-templated nucleotide residue at the 3′-end terminus of the antigenomic RNA strand. These results expand earlier observations on brome mosaic virus (BMV) related to the functional role of a non-templated 3′ terminal guanylate unique to the minus-strand RNA, which is required for efficient synthesis initiation by the BMV replicase. This appears to be the first characterization of such an RNA property *in vivo* for members of the genus.

## Data Summary

The Illumina datasets generated in this work are available in the public SRA repository under BioProject PRJNA1465229, with the following accessions: SRX33329027, SRX33329028, SRX33333591, SRX33333592, SRX33333742, SRX33333743, SRX33333744, SRX33333745, SRX33333746, SRX33333747, SRX33333748, SRX33333749, SRX33333750, SRX33333751, SRX33333752, SRX33333753, SRX33525604 and SRX33525605.

## Introduction

High-throughput sequencing (HTS) has revolutionized the discovery of novel viruses and the determination of viral genome sequences, providing a potent, rapid, unbiased approach for identifying viral agents and characterizing their genomes [1–5]. Through library preparation followed by bioinformatic analysis, it is nowadays possible to quickly reconstruct viral genome sequences, even from complex and low-abundance samples. Determination of the sequence of genome ends remains, however, challenging, as library preparation protocols are commonly unable to capture the terminal sequences in an effective manner. Because of this, data representing the 5′ and 3′ ends are typically insufficient, even at high sequencing depth [6–8]. This limitation becomes particularly critical when determining full-length genome sequences of novel viruses, especially those with highly divergent sequences and/or with multiple genome components.

Determination of the complete genome sequences of RNA viruses relies generally on RACE by PCR. The RACE method was first described by Frohman and colleagues [9], providing a strategy to amplify the cDNA ends using reverse transcription (RT) followed by PCR with gene-specific and adapter primers. RACE has since become a cornerstone technology for full-length cDNA cloning, transcript structure elucidation, investigations of UTRs, promoter activity and RNA processing events [10–13]. Two major variants of the technique exist. In the 5′ RACE, RT is initiated using a gene-specific primer (GSP), followed by the addition of a homopolymeric tail, or an adapter, to the 3′ end of the newly synthesized cDNA. This creates a priming site for anchor primers, enabling PCR amplification of the unknown 5′ sequence upstream of the internal primer-binding region. Instead, the classical 3′ RACE exploits the terminal poly(A) tail in the RNA. An oligo(dT) primer is used during RT, and PCR is performed using a sense GSP and an antisense primer binding the homopolymeric tail. Both versions typically utilize nested PCR strategies to enhance specificity, reduce background amplification and increase the final yield of the PCR product. In the absence of a poly(A) tail, a homopolymeric tail is added by RNA tailing to enable downstream reactions, with confidence in terminal sequence determination achieved through tailing with different ribonucleotides. This approach requires multiple reactions followed by Sanger sequencing of the resulting amplicons and relies on prior knowledge of viral genome sequence(s) for primer design. The procedure is time-consuming and labour-intensive, particularly for plant viruses with segmented genomes, where numerous PCR reactions substantially increase the experimental workload and complexity. In this context, complementary approaches that streamline RNA termini determination are highly desirable to improve and accelerate full-length sequence determination and genome characterization.

In the present study, we have optimized a commercial library preparation protocol [NEBNext^®^ Ultra^™^ II RNA Library Prep protocol for Illumina, New England Biolabs (NEB)] to enhance sequencing coverage of viral RNA termini and enable accurate reconstruction. The strategy is based on tailing of the 3′ terminal ends of the viral (genomic and antigenomic) RNA, followed by synthesis of cDNA using appropriate oligonucleotides and construction of a cDNA library (Fig. S1, available in the online Supplementary Material). The optimized strategy, which we named HTS-RARE (HTS-Rapid Analysis of RNA Ends), allows to sequence native 3′ ends, while 5′ ends of the genomic RNA are inferred from the 3′ terminal sequence of the antigenomic RNA. HTS-RARE was evaluated using reference plant RNA viruses from the collection at Leibniz Institute DSMZ, representing diverse taxonomic groups and genome organizations, and markedly accelerates and simplifies the determination of viral genome sequence ends directly from HTS data. Investigations in members of the genus *Ilarvirus* (family *Bromoviridae*) demonstrated the occurrence of a non-templated nucleotide at the 3′ end of the antigenomic RNA strand, absent in the plus-strand, supporting previous results obtained *in vitro* on the role of the additional nucleotide to promote efficient RNA synthesis initiation in brome mosaic virus (BMV; genus *Bromovirus*, family *Bromoviridae*), and suggesting that this feature may be common across members of the family.

## Methods

### Plant virus isolates

The plant virus isolates considered in this study belong to the reference collection at Leibniz Institute DSMZ (Braunschweig, Germany), and corresponding genome sequences are available in GenBank. The viruses selected for this study included the following: Belladonna mottle virus (BeMV), coyote tobacco stunting virus (CTSV), dandelion yellow mosaic virus (DaYMV), eggplant mottled dwarf virus (EMDV), pea enation mosaic virus 1 (PEMV1), pea enation mosaic virus 2 (PEMV2) and turnip yellows virus (TuYV), characterized by a monopartite genome; squash mosaic virus (SqMV), possessing a bipartite genome; asparagus virus 2 (AV2) and castor bean ilarvirus (CBIV), characterized by a tripartite genome; Mirafiori lettuce big-vein virus (MLBVV) and taro tenuivirus (TaTV), with a higher number of genomic RNA components. TuYV-associated RNA (TuYVaRNA), from accession PV-0995, was also considered in the analysis. Detailed information on each DSMZ PV accession, including virus taxonomic classification, genome properties, host plant used for propagation and GenBank accession number of the genome sequence, is summarized in Table 1.

**Table 1.** Accessions from the PV collection at Leibniz Institute DSMZ considered in this study

DSMZ accession	Virus common name/virus-associated RNA (acronym)	Virus species*	Genus	Genome composition	No. of genome segments	Propagation host	NCBI GenBank accession(s)
PV-0031	Eggplant mottled dwarf virus (EMDV)	*Alphanucleorhabdovirus melongenae*	*Alphanucleorhabdovirus*	ssRNA-	1	*Nicotiana rustica*	MW854257
PV-0042	Belladonna mottle virus (BeMV)	*Tymovirus belladonnae*	*Tymovirus*	ssRNA+	1	*Datura stramonium*	OR082757
PV-0427	Pea enation mosaic virus 1 (PEMV1)	*Enamovirus PEMV*	*Enamovirus*	ssRNA+	1	*Pisum sativum*	PP701462
PV-0427	Pea enation mosaic virus 2 (PEMV2)	*Umbravirus pisi*	*Umbravirus*	ssRNA+	1	*Pisum sativum*	PP701463
PV-0778	Dandelion yellow mosaic virus (DaYMV)	*Sequivirus taraxaci*	*Sequivirus*	ssRNA+	1	*Chenopodium quinoa*	OR762539
PV-0888	Mirafiori lettuce big-vein virus (MLBVV)	*Miraophiovirus mirafioriense*	*Miraophiovirus*	ssRNA-/ambisense	4	*Chenopodium quinoa*	PP856221–PP856224
PV-0956	Asparagus virus 2 (AV2)	*Ilarvirus AV2*	*Ilarvirus*	ssRNA+	3	*Nicotiana occidentalis*	PV870373–PV870375
PV-0995	Turnip yellows virus (TuYV)	*Polerovirus TUYV*	*Polerovirus*	ssRNA+	1	*Raphanus sativus*	PP503019
PV-0995	Turnip yellows virus-associated RNA (TuYVaRNA)	^†^	^†^	ssRNA+	1	*Raphanus sativus*	PP503020
PV-1054	Castor bean ilarvirus (CBIV)	^‡^	*Ilarvirus* (putative member)	ssRNA+	3	*Chenopodium quinoa*	PV664462–PV664464
PV-1120	Taro tenuivirus (TaTV)	^‡^	*Tenuivirus* (putative member)	ssRNA-/ambisense	5	*Nicotiana benthamiana*	PQ520508–PQ520512
PV-1178	Coyote tobacco stunting virus (CTSV)	^‡^	*Sobemovirus* (putative member)	ssRNA+	1	*Nicotiana benthamiana*	OR879103
PV-1424	Squash mosaic virus (SqMV)	*Comovirus cucurbitae*	*Comovirus*	ssRNA+	2	*Cucumis sativus*	PX094869–PX094870

*According to ICTV Master Species List MSL41.v1.

†Virus-associated RNA, not assigned to a genus.

‡Not listed in the ICTV Master Species List MSL41.v1.

### Preparation of total RNA extracts

Fresh tissue or lyophilized plant material sealed under vacuum in glass vials was used for isolation of total RNA using a number of commercial extraction kits, as detailed in Table S1.

### HTS-RARE

The HTS-RARE library preparation protocol developed in this study was established by adapting the protocol from the NEBNext^®^ Ultra^™^ II RNA Library Prep Kit for Illumina (NEB), with specific modifications to facilitate determination of RNA terminal ends in the subsequent bioinformatic analysis. Initially, total RNA extracted from plant leaf material (input ranging from 136 to 484 ng by quantification on NanoDrop spectrophotometer, Thermo Scientific) was subjected to 3′ terminal tailing with PAP [poly(A) polymerase, yeast; Thermo Fisher Scientific] and PUP [poly(U) polymerase; NEB], using the corresponding ribonucleotides. In the reaction, 3 µl of total RNA extract, 0.5 µl of RiboLock RNase Inhibitor (Thermo Fisher Scientific), 0.5 µl rATP or rUTP (25 mM stock solution) and 6 µl of water were carefully mixed, denatured for 3 min at 95 °C and thereafter cooled on ice. Following addition of the respective Master Mix, 1 µl RiboLock RNase, 1 µl PAP or PUP enzyme and water to a final total reaction volume of 25 µl, the tailing reaction was incubated at 37 °C for 11 min, followed by denaturation at 65 °C for 10 min. The tailed RNA was purified with a Monarch Spin RNA Cleanup Kit (10 µg) (NEB) and quantified with a NanoDrop spectrophotometer. Four microlitres of tailed RNA, with RNA quantity of 98 to 512 ng, were used as input for library preparation, exactly according to the manufacturer’s protocol, with the exception in the section described as ‘RNA Fragmentation and Priming’ (Section 4.1.1.; NEBNext^®^ Ultra^™^ II RNA Library Prep Kit for Illumina Manual Version 3.1_5/20). In the corresponding reaction, the random primers from the kit were replaced with 1 µl of a poly(A) or poly(T) primer (10 nt long, 25 mM working solution) (Table S2), according to the corresponding RNA tailing reaction. In addition, in Section 4.1.1 of the original protocol manual, a treatment with 0.5 µl of QIAseq FastSelect -rRNA Plant Kit (Qiagen) per reaction was implemented to allow depletion of the plant ribosomal RNA. Samples PV-0031, PV-0042, PV-0427, PV-0778, PV-0888, PV-0956, PV-1054 and PV-1178 were processed individually. In order to challenge the protocol with a complex biological sample, RNA extracts prepared from PV-0995, PV-1120 and PV-1424 were pooled (1 µl each) before the tailing reaction and thereafter processed as a bulked sample as described above.

### Sequencing of HTS libraries

HTS was performed at Leibniz Institute DSMZ on a NextSeq 2000 platform, to generate 2×150 bp paired-end reads. The number of raw reads generated for each sample is reported in Table S1, together with other data outputs from the bioinformatic pipeline.

### Bioinformatic analysis

All bioinformatic analyses were conducted in Geneious Prime v. 2026.0.2 (Dotmatics). To evaluate the efficiency of the tailing reaction in the HTS-RARE protocol, the ‘Map to Reference’ function with ‘Geneious’ mapper implemented in Geneious Prime was used to map the obtained reads to reference sequences consisting of mononucleotide stretches (150, 100, 50 and 30 nt in length), allowing 0 mismatches and 100% coverage along the reference. To determine the accordance of the viral end sequences as obtained with the HTS-RARE method to the PV reference sequences available in GenBank, the HTS reads were trimmed (error probability limit: 0.01) and aligned using the ‘Map to Reference’ function to the genome sequence available in GenBank (20% maximum mismatches allowed). The read alignment was performed independently for the PAP and PUP libraries of each accession in the study. Inspection of the alignments allowed determination of the ends for each RNA segment, considering a minimum of 3 nt as the length of the tail and a downstream sequence specific to the RNA segment. To count the number of tails covering each RNA end, the ‘motif search’ function implemented in Geneious Prime was thereafter used (0 mismatches) to search the mapped trimmed reads, with the motif consisting, as default, of 7 nucleotides specific to the virus sequence and at least 3, 7 or 20 mononucleotides. For viruses with RNA segments with conserved sequence ends, the minimum overlapping region was progressively extended until a unique nucleotide was identified; the total length of the region is reported in the ‘Minimum overlap’ column in Table S3.

### RACE reactions

For 3′ RACE reactions, the total RNA was tailed using PAP-Yeast (Thermo Fisher Scientific), in independent reactions with rATP, and/or rUTP, rCTP and rGTP ribonucleotides. The choice of the tailing reaction to conduct was based on analysis of the available sequence to minimize/avoid eventual pairing of the thereafter used primer in the amplification reaction to homopolymeric regions. Subsequently, cDNA synthesis was performed using an oligonucleotide primer composed of the complementary base (Table S2), and the WarmStart RTx Reverse Transcriptase kit (NEB), according to the manufacturer’s instructions. The PCR reactions were performed using the Q5 Hot Start High-Fidelity 2X Master Mix (NEB), with a GSP about 200–400 nt distant from the expected 3′ end (Table S2) and the appropriate reverse oligonucleotide with a homopolymer sequence complementary to the one used for cDNA synthesis. The PCR products were analysed on a Tris-EDTA agarose gel, and the products of expected size were purified with a Gel and PCR Clean-up kit (Macherey-Nagel) and sent directly for Sanger sequencing with the GSP or a nested primer appropriately designed (Microsynth).

For 5′ RACE reactions, two strategies were employed. In one approach (RACE from tailed cDNA), a cDNA synthesis reaction was performed with a GSP designed to bind 200–400 nt distant from the presumed 5′ end (Table S2), using the WarmStart RTx Reverse Transcriptase (NEB). The RNA/cDNA hybrid was made blunt-ended using a high-fidelity polymerase (Q5 Hot Start High-Fidelity 2X Master Mix, NEB), and the polished and purified cDNA (Gel and PCR Clean-up kit, Macherey-Nagel) was thereafter tailed using recombinant terminal deoxynucleotidyl transferase (Promega). The subsequent PCR reaction was performed with a nested GSP and the complementary primer to the homopolymeric tail. The second approach (RACE from tailed RNA) took advantage of the tailing of the antigenomic RNA strand in the 3′-end tailing reaction and RT, therefore generating a template for a PCR reaction with a GSP designed at the 5′ end (Table S2) and determination of the sequence end of the antigenomic RNA strand. In both strategies, the PCR products were applied on an agarose gel, and DNA amplicons of the expected size were purified and sent directly for Sanger sequencing with the GSP or an appropriate nested primer.

## Results

### Evaluation of the efficiency of the RNA tailing reaction implemented in the HTS-RARE protocol

**Fig. 1.**
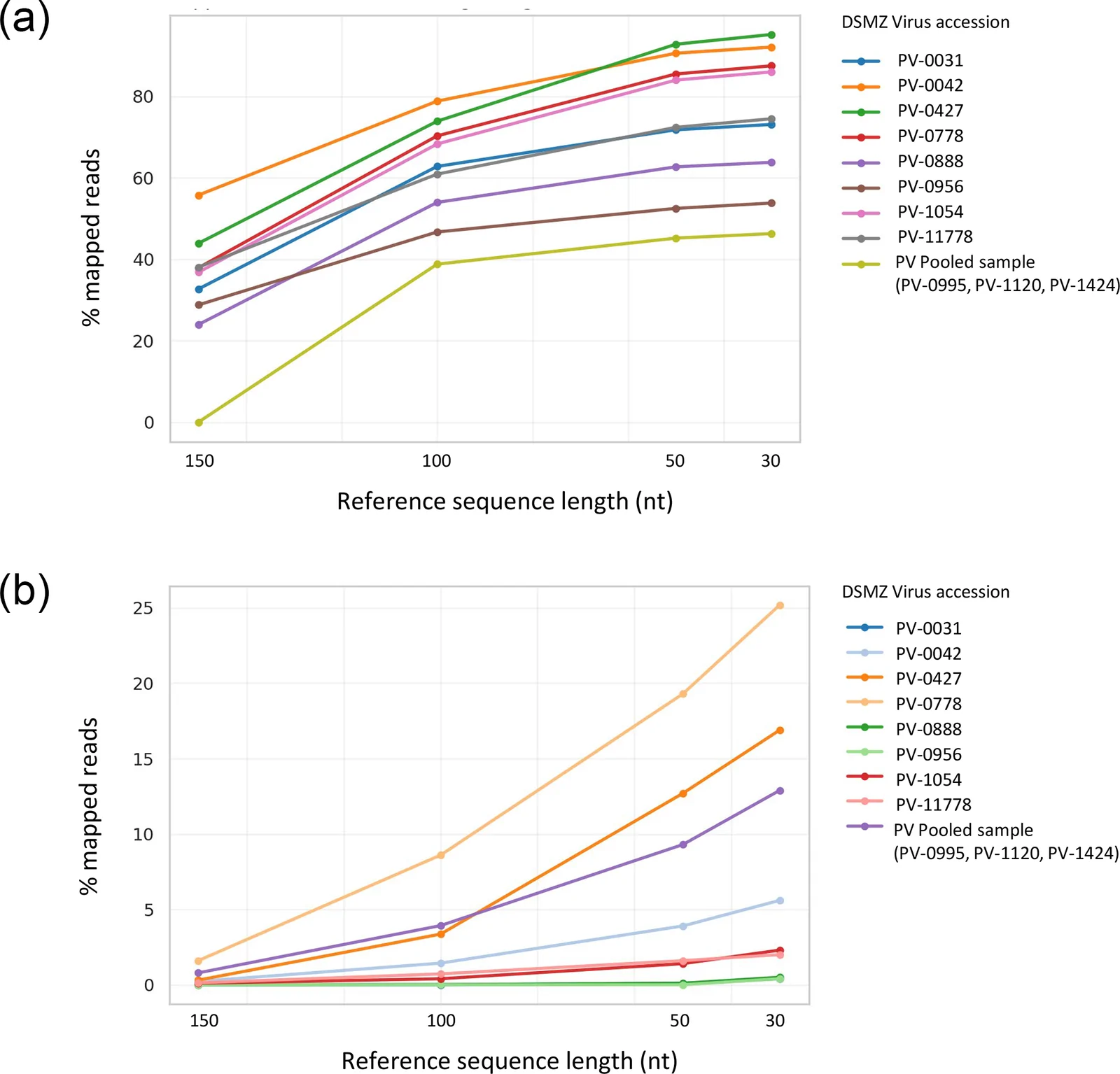
Evaluation of the efficiency of the RNA tailing reactions. (**a**) Graph showing the percent of mapped reads from poly(A)-tailing derived libraries to a reference sequence of defined length. (**b**) Graph showing the percent of mapped reads from poly(U)-tailing derived libraries to a reference sequence of defined length. The reference consisted of a mononucleotide stretch sequence of specific length (*n*=150, 100, 50 or 30 nt).

In total, nine libraries were prepared from PAP-tailed total RNA and nine from PUP-tailed total RNA. The number of generated reads per library ranged from ~1 Mio up to ~19 Mio (Table S1). To verify the efficiency of the RNA tailing reactions and the effectiveness of the procedure, a first analysis was conducted by counting the number of reads harbouring a homopolymeric tail (A/T) of defined length, at four cut-off values (at least 150, 100, 50 and 30 nt long) (Table S1). In the PAP-derived libraries, 21–55% of the raw reads were at least 150 nt in length, with repetitive A or T residues (Table S1, Fig. 1). In contrast, in the PUP-derived libraries, the percentage of reads with at least 150 nt-long repetitive A or T nt accounted for less than 0.1% up to 1.6% (Table S1, Fig. 1), showing a superior performance of PAP-tailing reactions. Examination of reads with shorter mononucleotide tails resulted in an increase in the percentage of reads, as expected, and validated the initial observation (Fig. 1). In a comparison with datasets from libraries prepared from genuine (not tailed) RNA, the percentage of sequence reads with repetitive A or T residues was in all cases less than 0.1% (Table S1), showing that the tailing reactions implemented in the HTS-RARE protocol were efficiently conducted and that the homopolymeric stretches were for the majority, a product of the tailing reactions.

### Determination of RNA terminal sequences via HTS-RARE

**Fig. 2.**
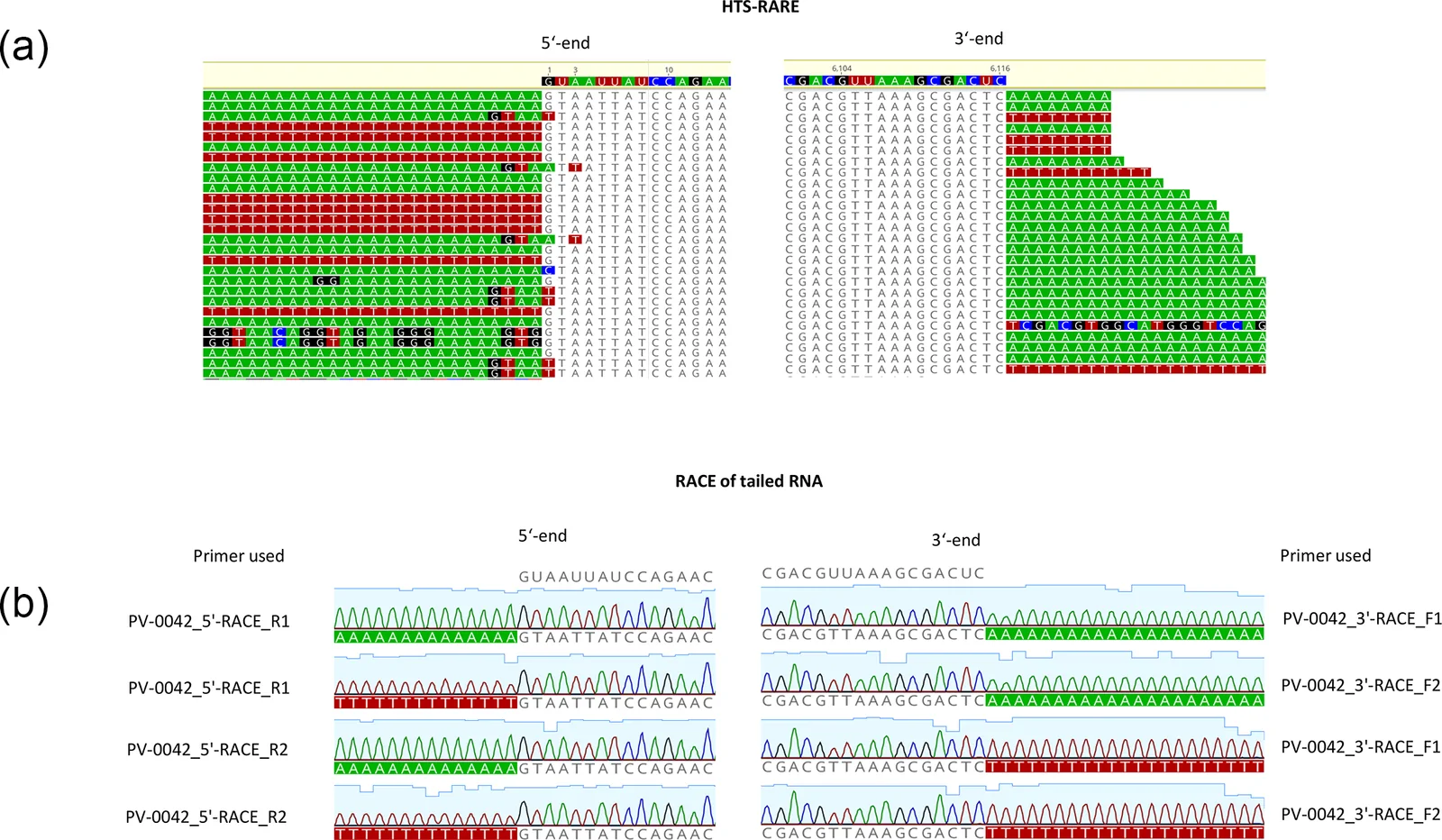
Representative comparison of HTS-RARE output and classical RACE of tailed RNA. (**a**) Screen capture of alignment of the sequence reads obtained by HTS-RARE to the terminal sequences of the genomic RNA of accession PV-0042, BeMV (GenBank reference sequence OR082757). It should be noted that the 5′ homopolymeric sequences shown derive from homopolymeric sequences appended to the 3′ end of native antigenomic RNAs in the sample. (**b**) Chromatograms obtained by Sanger sequencing of RACE amplicons generated by tailing of the viral RNA with PUP and PAP polymerase, and amplification using distinct virus-specific primers per tailing reaction.

For evaluation of the performance of the HTS-RARE strategy, 12 viruses and an associated viral RNA were considered (Table 1). This resulted in a total of 25 RNA segments, whose sequence ends could be successfully reconstructed. Results are presented in Table 2 with a colour coding for easier interpretation of the results related to the number of distinct tails per each RNA end, as follows: (i) if both PAP and PUP-derived tails were found at a RNA sequence end, the sequence was considered doubly validated (case marked as dark grey); (ii) if only one of the tails was found, then the first true residue could be the same as the one of the tail, and the case was marked as light grey. Overall, of the 50 RNA sequence ends, 44 could be determined with 2 independent tails (Table 2). For 6 RNA segments, the 5′ terminal end could be determined with only one tail (light grey). A representative alignment of the HTS-RARE sequence reads to a reference sequence is provided in Fig. 2, benchmarked with the results obtained via a classical RACE reaction, showing 100% accordance between the two methods.

**Table 2.** Overview of the RNA sequence ends of the accessions in the study determined via the HTS-RARE method

DSMZ accession	Virus/virus-associated RNA*	Segment	GenBank accession	HTS-RARE		Final curated sequence		HTS-RARE	
				**5′ end**				**3′ end**	
				**T-tail**	**A-tail**	**7 nt terminus 5′ end**	**7 nt terminus 3′ end**	**A-tail**	**T-tail**
PV-0031	EMDV		MW854257	+	+	ACACACC…	…GGUGUGU	+	+
PV-0042	BeMV		OR082757	+	+	GUAAUUA…	…GCGACUC	+	+
PV-0427	PEMV1		PP701462	+	+	GUGAAAU…	…AUAUCGC	+	+
PV-0427	PPEMV2		PP701463	+	+	GGGUAUU…	…GGCGCCC	+	+
PV-0778	DaYMV		OR762539	+	+	GUUAAAA…	…AUAAGGC	+	+
PV-0888	MLBVV	RNA 1	PP856221	+	+	GAUAUAU…	…AAUAAUC	+	+
	MLBVV	RNA 2	PP856222	+	+	GAUAUUU…	…AAUAAUC	+	+
	MLBVV	RNA 3	PP856223	+	+	GAUAGUU…	…AAUAAUC	+	+
	MLBVV	RNA 4	PP856224	+	+	GAUAUUU…	…AAUAAUC	+	+
PV-0956	AV2	RNA 1	PV870373	+	−	(C)GUAUUGU…†	…GAGAUGC	+	+
	AV2	RNA 2	PV870374	+	−	(C)GUAUUGU…†	…GAGAUGC	+	+
	AV2	RNA 3	PV870375	+	−	(C)GUAUUCA…†	…GAGAUGC	+	+
PV-0995	TuYV		PP503019	+	+	ACAAAAG…	…UCGGUGU	+	+
PV-0995	TuYVaRNA		PP503020	+	+	GGGAUUU…	…ACCGCCC	+	+
PV-1054	CBIV	RNA 1	PV664462	+	+	(C)GUAUUGU…†	…GAGAUGC	+	+
	CBIV	RNA 2	PV664463	+	−	(C)GUAUUGU…†	…GAGAUGC	+	+
	CBIV	RNA 3	PV664464	+	−	(C)GUAUUCA…†	…GAGAUGC	+	+
PV-1120	TaTV	RNA 1	PQ520508	+	+	ACACAAA…	…UAUGUGU	+	+
	TaTV	RNA 2	PQ520509	+	+	ACACAAA…	…UUUGUGU	+	+
	TaTV	RNA 3	PQ520510	+	+	ACACAAA…	…UUUGUGU	+	+
	TaTV	RNA 4	PQ520511	+	+	ACACAAA…	…UUUGUGU	+	+
	TaTV	RNA 5	PQ520512	+	+	ACACAAA…	…UAUGUGU	+	+
PV-1178	CTSV		OR879103	+	+	ACAAAAU…	…GGUGAGU	+	+
PV-1424	SqMV	RNA 1	PX094869	+	−	UAUUAAA…	…UCUUUUG(A_n_)	+	+
	SqMV	RNA 2	PX094870	+	+	UAUUAAA…	…UCUUUUU(A_n_)	+	+

*Refer to Table 1 for acronyms.

Characters and colours legend: +, tail found; −, no tail found.

RNA sequence end determined with reads generated by sequencing of libraries from PAP and PUP tailing reactions.

RNA sequence end determined with the library from one tailing reaction.

†Residue in brackets was determined by HTS-RARE to be present only in the antigenomic RNA as guanylate residue.

(An), natural poly(A) tail of the viral RNA.

By comparing the coverage, we observed that, overall, the total number of sequencing reads mapping a reference mononucleotide sequence was generally lower at the 5′ end compared to the 3′ end (Table S3). The 5′ end of the positive-sense RNA strand appeared instead more covered for viruses with a negative/ambisense genome, as per PV-0031, PV-0888 and PV-1120 (Table S3). This likely correlated with the antigenomic RNA strand being much less abundant in the total RNA extraction than the genomic RNA strand for positive-sense RNA viruses. Success was also observed in the pooled sample composed of three PV accessions (PV-0995, PV-1120 and PV-1424), where 5′ and 3′ sequence ends could be determined for all RNAs in the study, therefore allowing tailing, sequencing and successful analysis of a complex sample from a single library preparation (Table 2).

#### Comparison of sequences obtained by HTS-RARE to GenBank reference entries

**Fig. 3.**
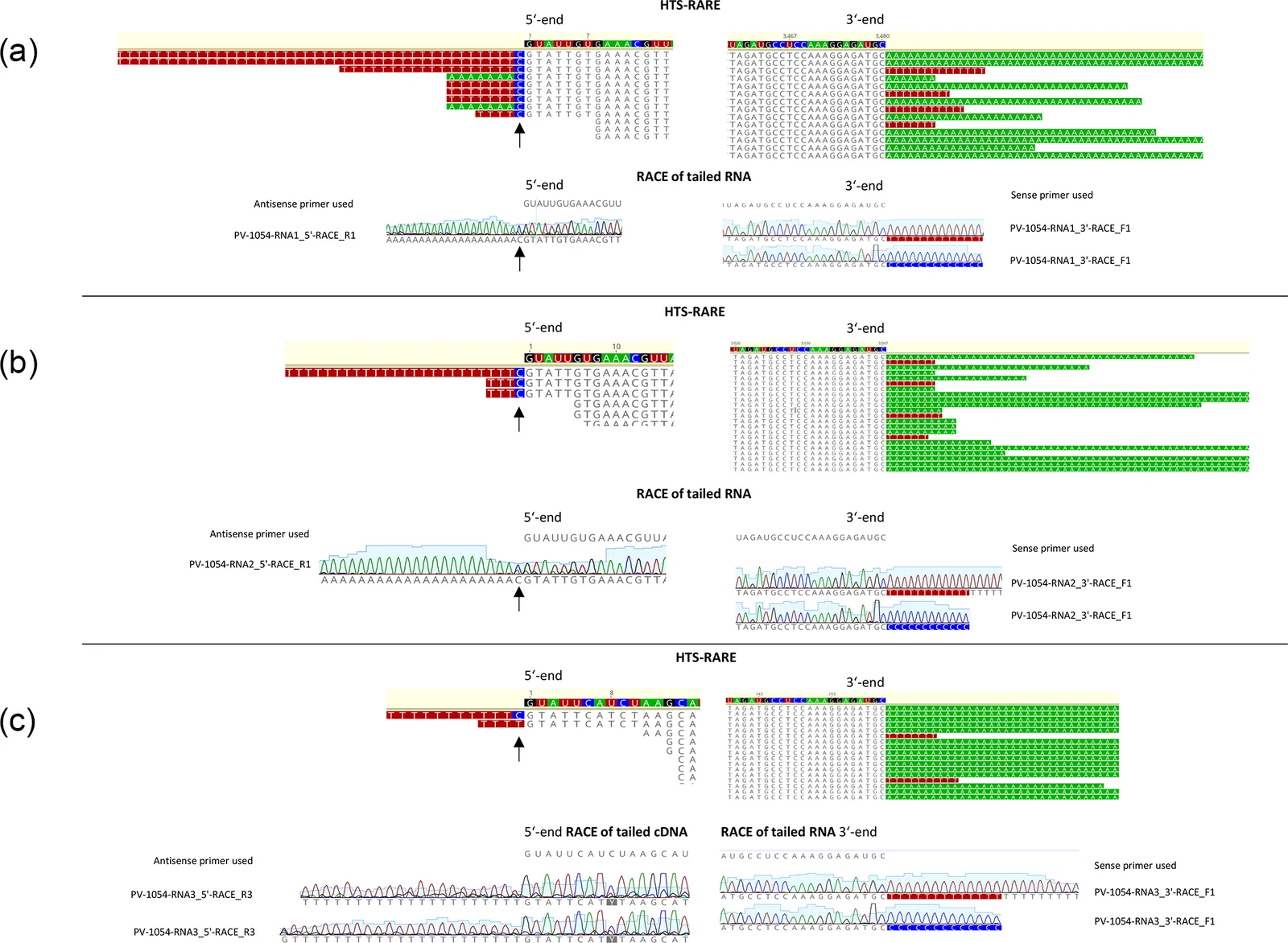
Results obtained for accession PV-1054, CBIV. (**a**) HTS-RARE reads and chromatograms obtained by RACE from tailed RNA1, aligned to the GenBank reference sequence PV664462 (PV-1054 RNA1). (**b**) HTS-RARE reads and chromatograms obtained by RACE from tailed RNA2, aligned to the GenBank reference sequence PV664463 (PV-1054 RNA2). (**c**) HTS-RARE and chromatograms obtained by RACE from tailed RNA or from tailed cDNA reverse-transcribed from RNA3, aligned to the GenBank reference sequence PV664464 (PV-1054 RNA3). The black arrows point to the cytidylate residue, revealed by tailing of the antigenomic RNA strand.

Comparison of the 50 RNA sequence ends obtained via HTS-RARE to the sequence of the PV accessions available in GenBank, determined via standard RACE or via prediction by comparison to sequences of other isolates of the same virus or closely related members and subsequent validation by inspection of read alignment, revealed that most of them (40) were the same. Differences were revealed for 10 RNA ends (all corresponding to the 5′ end; Table 2) of three PV accessions: the three-segmented PV-0956 (AV2) and PV-1054 (CBIV; Fig. 3), accepted and putative member of the genus *Ilarvirus*, respectively, and the four-segmented PV-0888 (MLBVV) in the genus *Miraophiovirus*.

For PV-0956 and PV-1054, the HTS-RARE determined the presence of an additional guanylate at the 3′ terminus of the antigenomic RNA strand, therefore resulting in a putative cytidylate residue at the 5′ terminus of the genomic RNA sequence, which was not recorded in the GenBank reference entry obtained by sequencing of cDNA libraries prepared according to the protocol instructions of the kit (Table 2 and S3). The opposite case was observed for PV-0888: here, the HTS-RARE revealed no adenylate residue at the 3′ terminus of the antigenomic RNA strand, while a uridylate residue was present at the 5′ terminus of each of the four RNA segments in the MLBVV reference accessions (GenBank accession nos. AF525933 to AF525936) and in RNA2, RNA3 and RNA4 of the originally determined sequence for PV-0888 (Table S3). These results stimulated further investigations, which are presented below.

#### Occurrence of a non-templated nucleotide addition at the extreme 3′ end of the viral complementary RNA strand of members of the genus *Ilarvirus*

Considering the disagreement of the 5′ sequence ends of the analysed ilarviruses, and based on the consideration that by using the HTS-RARE strategy, the antigenomic RNA strand is tailed at the 3′ end, and that the sequence of the genomic RNA strand at the 5′ end is inferred rather than determined directly, we investigated whether the additional cytidylate was a non-templated nucleotide of the antigenomic RNA strand, not indeed present in the genomic RNA strand. For this purpose, the accession PV-1054 was considered for two RACE approaches. In the first, when antigenomic RNA was tailed and thereafter used for RT, the cytidylate residue was confirmed at the 3′ end of the tailed minus-strand RNA, in accordance with what was observed via HTS-RARE (Fig. 3). In the second RACE approach, when cDNA was synthesized from the genomic RNA strand, tailed and thereafter amplified, Sanger sequencing of the obtained amplicon showed instead the absence of a guanylate residue in the same position in the tailed cDNA (Fig. 3). Overall, for both ilarviruses, the HTS-RARE strategy allowed to prove the occurrence of a guanylate extra residue that is unique to the 3′ end of the antigenomic RNA, and absent in the derived genomic strand.

#### Verification of the 5′-end terminal sequence of the genomic RNA strands of MLBVV

**Fig. 4.**
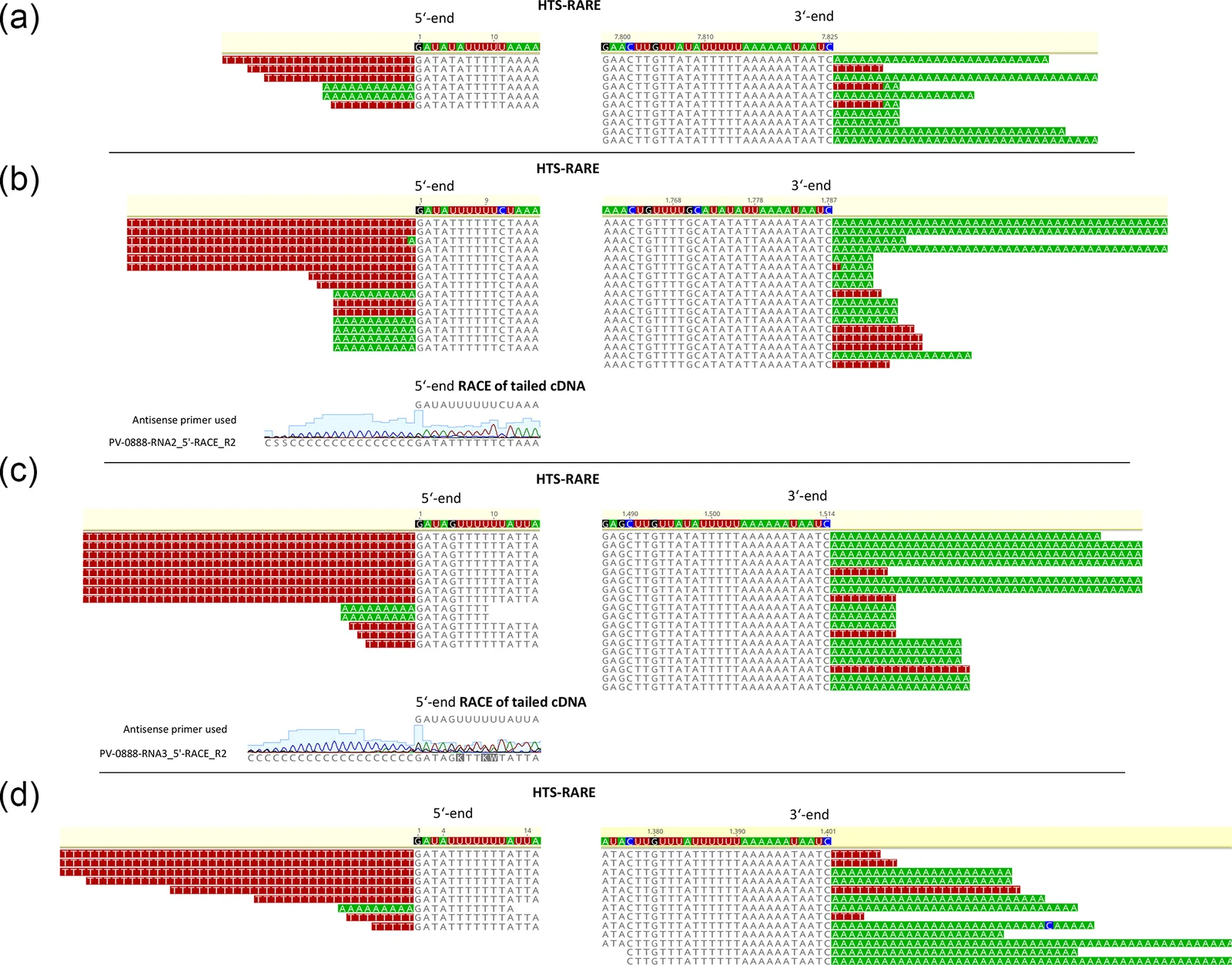
Results obtained for accession PV-0888, MLBVV. (**a**) HTS-RARE reads, aligned to the finally determined RNA1 sequence of PV-0888. (**b**) HTS-RARE reads and chromatogram obtained by 5′ RACE from tailed cDNA reverse-transcribed from RNA2, aligned to the finally determined RNA2 sequence of PV-0888 RNA2. (**c**) HTS-RARE reads and chromatogram obtained by 5′ RACE from tailed cDNA reverse-transcribed from RNA3, aligned to the finally determined RNA3 sequence of PV-0888. (**d**) HTS-RARE reads, aligned to the finally determined RNA4 sequence of PV-0888.

Considering the disagreement observed at the 5′ end of the RNA sequences of the miraophiovirus, we investigated whether the additional uridylate residue present in the PV accession sequence (Table 1) was a derived by-product of the bioinformatic analysis by comparison to the GenBank reference sequence, not present *in vivo* in the genomic strand. For this purpose, the accession PV-0888 was considered for a RACE approach, including synthesis and blunt-ending of the cDNA, followed by tailing and amplification. The sequence analysis showed that no additional uridylate residue was present at the 5′ end of the successfully processed RNA2 and RNA3 (Fig. 4).

## Discussion

### A novel assay for determination of viral RNA ends

The advent of HTS technologies has accelerated the reconstruction of viral genome sequences. However, the ability to effectively capture the terminal sequence ends still remains challenging. Former studies have aimed to address this issue using modern sequencing technologies to simplify reconstruction of terminal end sequences compared to conventional techniques, such as RACE. A RACE-Seq methodology combining massive parallel sequencing with 5′ RACE had been established in 2009 to overcome the limitation in determining terminal sequence information and verify transcriptional starting sites [14]. The method still relied on standard RT and short-read sequencing, which can generate truncated products and limit accurate resolution of complex 5′ UTR structures. A 5′ RACE-Seq assay [15] incorporated optimized template switching during RT to preferentially capture full-length, capped mRNAs and paired this strategy with long-read nanopore sequencing, enabling more precise identification of transcription start sites and detection of alternative 5′ UTR variants. In virology, a study from Alfons and colleagues [6], described a streamlined next-generation sequencing approach involving depletion of polyadenylated RNA, random fragmentation and adapter ligation directly to RNA, for obtaining complete viral genome sequences of Ebola and hepatitis C virus (HCV), including terminal regions, using a single HTS run. In our study, we have established a new strategy that allows rapid determination of viral RNA terminal sequence ends, by fine-tuning a standard and well-established HTS library preparation protocol to enhance their recovery. The method was validated on 12 accessions representing virus members of distinct genera, along with a virus-associated RNA (Table 1), encompassing in total 25 RNA segments. In this context, determination of the 5′ and 3′ terminal sequences, each by two independent tailing reactions using standard RACE, would require an impractical number of tailing, amplification and Sanger sequencing reactions, making this approach highly time-consuming and almost prohibitive. The library preparation kit considered for implementation of the protocol and validation was the NEBNext^®^ Ultra^™^ II RNA Library Prep Kit for Illumina (NEB), which enables non-strand-specific RNA library construction with significantly improved yield and sensitivity compared to previous-generation kits. The protocol is compatible with both poly(A) mRNA enrichment and rRNA depletion workflows and supports a wide input range, from as little as 10 to 1 µg of total RNA. Overall, HTS-RARE was successful in determining the RNA sequence ends, with considerable efficiency. Our results show that its implementation effectively alleviated the efforts necessary in the bioinformatic analysis to determine the sequence of terminal RNA regions.

A primary advantage of the HTS-RARE method when determining sequence ends relies on the high sequence depth obtained by sequencing, with up to several 10,000 reads at one RNA end (Table S3), compared to the consensus sequence obtained by traditional RACE and Sanger sequencing. Owing to the high number of reads, analysis of the sequence ends is much more meaningful, and it is also possible to look for variants that are present in low numbers. Further, traditional RACE approaches are challenging in the RT reaction or PCR amplification phase. For example, when the 3′ end of an RNA is A-rich, generating an RT-PCR fragment using a poly(T) primer can be challenging, due to the mis-annealing within the A-rich regions and eventual preferential amplification of the shorter region. Although RT and PCR are also part of the HTS-RARE library preparation protocol, the PCR amplification has a minimum number of cycles (generally 9, based on Section 6.3.3.; NEBNext^®^ Ultra^™^ II RNA Library Prep Kit for Illumina Manual Version 3.1_5/20), and the high number of generated reads allows then to cover the whole target region.

We observed a higher efficiency of the PAP tailing reactions compared to the PUP tailing. In the comparison of efficiency, 21–55% of all reads in the PAP library contained an ‘A’ stretch of at least 150 nt, while for PUP libraries, the amount was significantly lower. In addition, six of the tails that were not found (Table 2) were 5′ ‘A’-tails, which should be generated from the PUP tailing reaction. This result shows that PAP tailing is more effective than PUP tailing when using the method described here. In an initial attempt, we also considered tailing with cytidylate and guanylate ribonucleotides; however, efficiency was unsatisfactory (not shown). According to the manufacturer, the PUP enzyme can add only a limited number (~5–20) of guanylate residues to the 3′ end of RNA, and PAP shows strong substrate preference for rATP over rGTP, rCTP or rUTP. The resulting short G- or C-rich tail (<20 nt) is likely then to be insufficient to support efficient library construction. Therefore, PAP and PUP tailing with the respective ribonucleotides remains the best strategy for the two RNA tailing reactions in the HTS-RARE protocol.

A challenge in the use of HTS-RARE may arise when determining the exact genome termini for segmented viruses with highly similar sequence ends, specifically when the length of the conserved sequences exceeds the maximum length of the reads generated by sequencing. To overcome this limitation, a specific bioinformatic approach is needed to resolve the sequence of each RNA, by mapping the reads to a reference sequence extended up to an RNA-specific discriminatory nt. This remains critical for viruses harbouring highly conserved segment 3′ ends spanning several hundred nucleotides, e.g. nepoviruses [16–18], resulting in a challenge for exact sequence end determination.

As an additional strategy to reduce sequencing costs, we evaluated the pooling of RNA samples prior to library preparation, allowing multiple viral RNAs to be processed under a single barcode. This multiplexing approach was successfully implemented and did not compromise sequence recovery of the nine RNA segments, demonstrating its feasibility for cost-effective, high-throughput characterization of virus sequences. A further level of pooling, by combining PAP- and PUP-tailed RNAs as input in a single library preparation, was not tested; however, as the respective primers in the cDNA synthesis reactions are likely to interfere, such an approach would likely compromise library preparation and downstream analyses. As part of the study, we also assessed the feasibility of recovering full-length virus genome sequences via *de novo* assembly from HTS-RARE read datasets, compared to a standard library protocol. Evaluation of the resulting viral contigs from the respective assemblies revealed, however, that those generated from the HTS-RARE datasets were generally substantially shorter and more abundant than those from the standard library (Table S4). This result suggests that *de novo* assembly under these conditions would be considerably more complex, requiring a combination of standard library and HTS-RARE to recover the full-length RNA sequence.

#### HTS-RARE provides evidence of an RNA feature across members of the genus *Ilarvirus*

An interesting finding common to the two ilarviruses investigated in this study was the occurrence of a non-templated residue in the antigenomic RNA. This finding stimulated further investigations via standard RACE to verify the finding. Indeed, in a former study on BMV (genus *Bromovirus*, family *Bromoviridae*), by using DNA templates to define the requirements for RNA-dependent RNA polymerase (RdRP) template recognition, the authors reported that efficient initiation from the 3′ end of the template required the presence of an additional nucleotide 3′ of the initiation site [19]. Furthermore, addition of a non-templated nucleotide to the 3′ end of BMV minus-strand RNAs enabled efficient initiation of genomic plus-strand RNA synthesis *in vitro*. These results supported that the presence of a specific 3′-terminal nucleotide is a key feature for BMV RNA replication, pointing that the viral replicase complex can generate or select RNA ends optimized for *de novo* synthesis initiation of the genomic RNA. Notably, in our study, we proved the occurrence *in vivo* of non-template guanylate residues at the 3′ terminus of the RNA minus strand of ilarviruses, also members of the *Bromoviridae*. We showed this property for CBIV (PV-1054) and AV2 (PV-0956), and the specific feature was common to all RNA components. This finding was confirmed via a complementary approach, by tailing the antigenomic RNA strand followed by RT-PCR and Sanger sequencing (Fig. 3). As corroboration of the findings, when cDNA was made from the genomic strand and the cDNA/RNA hybrid was made blunt-ended and then amplified and sequenced, no cytidylate was present, proving that the feature was specific to the minus-strand RNA (Fig. 3).

Of note, non-templated terminal nucleotide addition by viral RdRPs has been reported in several animal and human RNA viruses, indicating that this activity reflects a broader, intrinsic property of viral polymerases. Biochemical studies on HCV and bovine viral diarrhoea virus demonstrated that highly purified RdRPs possess an intrinsic terminal nucleotidyltransferase activity, which enables template-independent addition of one or more nucleotides to the 3′ end of RNA molecules [20, 21]. Non-templated 3′-end extensions have been reported for RdRPs from noroviruses, polioviruses and bacteriophage φ6, demonstrating that viral RdRPs can deviate from strict template-directed synthesis to modify RNA termini [22–24]. Overall, in light of the results presented in our study, RdRPs with such a property appear to occur in the plant virus infections examined. We speculate that this mechanism could constitute an additional layer of control in positive-strand RNA virus replication mediated through 3′-end RNA modifications. Our findings stimulate further investigations to determine whether similar properties are shared by virus groups whose replication machinery/RdRPs are more closely related to that of *Bromoviridae*.

#### HTS-RARE results stimulate considerations on previous sequence assemblies

For most considered viruses, the sequence ends determined via HTS-RARE corresponded to the ones from the classical approach. The distinctive cases prompted further investigations, yielding noteworthy results. With HTS-RARE, we were able to show that a uridylate is not the initial residue at the 5′ end of the RNA segments of MLBVV, contrary to the previously determined sequences [25]. To be noted here is that with the HTS-RARE method, the 5′ end is determined on the basis of the tailing of the antigenomic strand. In contrast, in the RACE method from tailed cDNA, cDNA is first synthesized and then tailed using recombinant terminal deoxynucleotidyl transferase. It has been described that Moloney murine leukaemia and avian myeloblastosis virus reverse transcriptases can add non-template adenylate residues to the cDNA [26]. As a result, if the cDNA/RNA hybrid is not made blunt-ended before proceeding, the non-template adenylate residue can cause the 5′ end to be determined incorrectly. We suspect that this was the case with MLBVV [25], where a classical 5′ RACE was conducted, and the same may have happened for pepper chlorosis-associated virus [27], which belongs to the same genus. Indeed, the reference sequences for MLBVV PV-0888 (GenBank accessions PP856221, PP856222, PP856223 and PP856224), obtained via standard library preparation (Table S1), have been described as starting ‘UGAUA’ at the extreme 5′ end. To be noted is that the reference sequence ends were originally reconstructed based on comparison with the original deposited sequences from a former study (AF525933–AF525936), where genome terminal sequences were determined via 5′ RACE by tailing cDNA with dCTP [25]. With the HTS-RARE approach, the first uridylate residue could not be confirmed for any of the four RNA genomic segments (Fig. 4), and this was verified via a 5′ RACE on a blunt-ended cDNA/RNA hybrid.

#### Prospective applications of HTS-RARE

Besides being required for precise characterization of molecular features of RNAs, we foresee that the presented strategy can offer significant advantages in the generation of high-quality reference genome sequences. Of note, this could be especially valuable in cases when infectious clone systems are to be established and accurate reconstruction of the complete viral genome, including the precise 5′ and 3′ terminal sequences, is desired. Indeed, it has been documented that even minimal alterations at genome termini, such as the loss or addition of a few nucleotides, can abolish infectivity, as documented for tobacco mosaic virus, turnip crinkle virus and tomato chlorosis virus [28, 29]. We foresee that HTS-RARE can enhance the accuracy of terminal sequence determination during genome assembly and cloning, thereby increasing the reliability and success of generating fully functional infectious clones to investigate molecular features and virus biological properties.

## Conclusion

We optimized a standard RNA library preparation protocol to improve sequencing coverage of viral RNA termini and enable accurate determination of 5′ and 3′ ends. Assessment of performance on analysis of diverse plant RNA viruses and benchmarking against a classical library construction strategy demonstrated that HTS-RARE successfully accelerates and simplifies terminal sequence identification directly from HTS data. This method offers a robust alternative to labour-intensive RACE approaches and can be applied broadly in diverse research fields for the characterization of properties of RNA molecules.

## Supplementary material

10.1099/jgv.0.002331 Fig. S1.

10.1099/jgv.0.002331 Supplementary Material 1.

## Abbreviations

AV-2: asparagus virus 2
BeMV: Belladonna mottle virus
BMV: brome mosaic virus
CBIV: castor bean ilarvirus
CTSV: coyote tobacco stunting virus
DaYMV: dandelion yellow mosaic virus
EMDV: eggplant mottled dwarf virus
GSP: gene-specific primer
HCV: hepatitis C virus
HTS: high-throughput sequencing
HTS-RARE: High-Throughput Sequencing-Rapid Analysis of RNA Ends
MLBVV: Mirafiori lettuce big-vein virus
NEB: New England Biolabs
PAP: poly(A) polymerase
PEMV1: pea enation mosaic virus 1
PEMV2: pea enation mosaic virus 2
PUP: poly(U) polymerase
PV: plant viruses
RACE: rapid amplification of cDNA ends
RdRP: RNA-dependent RNA polymerase
RT: reverse transcription
SqMV: squash mosaic virus
TaTV: taro tenuivirus
TuYV: turnip yellows virus
TuYVaRNA: TuYV-associated RNA
UTR: untranslated region
