# Supplementary material for: Enhanced recovery of terminal sequences of viral RNAs via fine-tuning of the high-throughput sequencing library preparation and evidence of a non-templated nucleotide addition at the 3′ end of the minus strand in members of the genus Ilarvirus
Source: J Gen Virol. 2026 Sep 3;107(9):002331. doi: 10.1099/jgv.0.002331 (PMC13541289; doi:10.1099/jgv.0.002331)

Supplementary Figure S1 Graphical overview of the HTS-RARE workflow

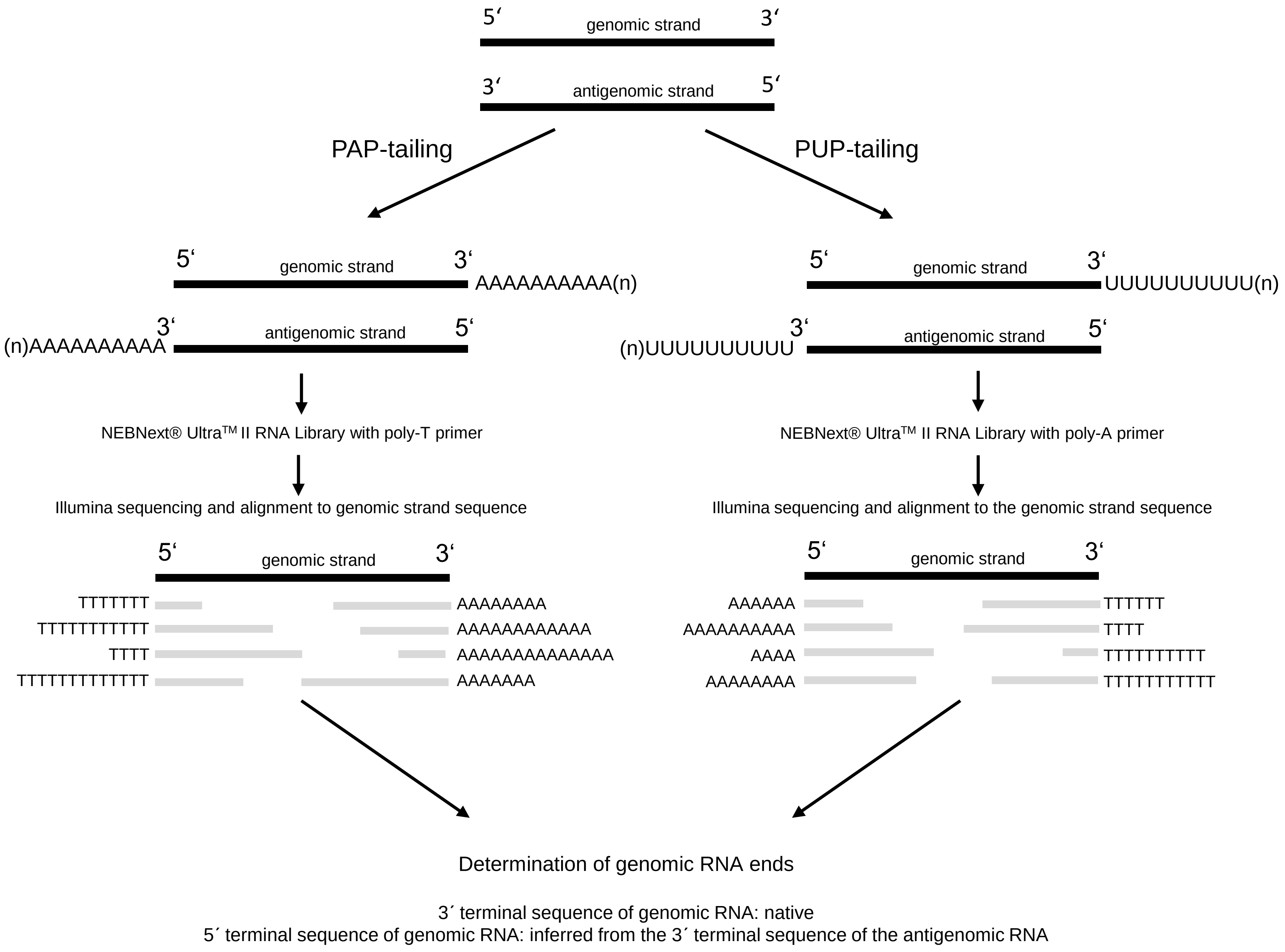

Supplement: Supplementary file 1 [file jgv-107-02331-s001.pdf]
